# Immunological outcomes of Tenofovir versus Zidovudine-based regimens among people living with HIV/AIDS: a two years retrospective cohort study

**DOI:** 10.1186/s12981-017-0132-4

**Published:** 2017-02-01

**Authors:** Teshale Ayele, Habtemu Jarso, Girma Mamo

**Affiliations:** 1 Department of Pharmacy, Jimma University Medical Centre, Institute of Health, Jimma University, Jimma, Ethiopia; 2 Department of Epidemiology, Public Health Faculty, Institute of Health, Jimma University, Jimma, Ethiopia

**Keywords:** Immunological outcomes, Tenofovir regimen, Zidovudine regimen, Ethiopia

## Abstract

**Background:**

Tenofovir (TDF) based regimen was reported to have better immunological outcomes. Unfortunately, there is limited information regarding the immunologic outcome associated with this regimen in Ethiopia, as its routine utilization in this setting begun since 2013.

**Methods:**

A 2 years retrospective cohort study was conducted at Jimma University Specialized Hospital, 346 km Southwest of Addis Ababa, Ethiopia. A total of 280 patients’ data from September 2012 to July 2014 was extracted from records from February 10, 2015 to March 10, 2015. Records were selected using a simple random sampling technique. Data on socio-demographic, clinical and drug related variables were collected; entered into EpiData 3.1 and analyzed by STATA 13.1. Mixed effect linear regression was performed to assess difference in CD4+ change between groups adjusting for baseline characteristics. The change in predicted CD4 count attributed to each regimen was also assessed by marginal analysis. *P* < 0.05 for slopes of the random effect linear regression was used as indicators for presence of association.

**Results:**

The mean (SD) duration of cohort follow up was 714.2 (69.6) and 708.8 (78.9) days (*P* = 0.753) for TDF and AZT groups respectively. The minimum follow up duration was 7.4 and 8.9 months for TDF and AZT groups respectively. Most of TDF (93.6%) and AZT (91.4%) groups completed their follow up, 5 (3.6%) TDF and 6 (4.3%) AZT groups died and 4 (2.9%) TDF and 6 (4.3%) AZT groups were lost for follow-up (*P* = 0.769). There was statistically significant difference in immunologic recovery between the groups (B = +34.08, 95% CI [7.8, 60.35], P = 0.027) over time. The predicted CD4+ count for TDF/3TC/EFV was (B = +347.65 cells/mm^3^, P < 0.001) whereas that of AZT/3TC/EFV was (B = +281.54 cells/mm^3^, P < 0.001).

**Conclusions:**

TDF based regimens have shown more efficacy compared to AZT based regimens though AZT based regimens are more affordable in low income countries like Ethiopia. However, we recommend further study with quality design to assess the prevalence of sub-optimal CD4+ response (net CD4 gain <50 cells/µl/6 month) in this set-up among TDF users.

## Background

Around 1980s, Acquired immunodeficiency syndrome (AIDS) was globally emerged as a major public health threat. The reaction against it has led to unprecedented attention and commitment from the international community to improve access to human immune virus(HIV) care, antiretroviral treatment (ART) and prevention [1, 2]. The introduction of potent ART has dramatically reduced HIV/AIDS associated crisis—reduced rates of mortality and morbidity, improved quality of life, revitalized communities and transformed perceptions on the disease from a plague to a manageable chronic illness [3–6].

Currently, there are more than 20 ARV compounds approved for use in United States (US) and Europe. Multiple adult HIV treatment guidelines recommend the nucleos(t)ide reverse transcriptase inhibitors; AZT or TDF based regimens and most are in favour of TDF based regimens as the safer regimen for patients with no contraindication due to its proven effectiveness, favourable toxicity profile, and demonstrated regimen durability [1, 7–9].

Safe and efficacious ART regimens improve patient care through rapidly restoring the immune cells, promoting adherence and alleviating the hazards of mortality [1, 10, 11]. TDF is one of the ART drugs that come to be routinely utilized in Ethiopia since the past 2 years. For this drug, the recent WHO report indicated that data on the risk of major clinical events such as mortality, renal failure and, bone fractures were limited, and particularly for our setups there is no immunologic finding as well [11].

Studies from developed regions showed that there were 18.0 and 18.8% immunologic failures in the EFV/FTC/TDF and EFV/3TC/AZT arms, respectively. There were no significant differences in the risk of HIV-1 disease progression or death [12]. In other findings, TDF based regimens has demonstrated a better immunologic outcome only when combined with Efavirenz as compared to AZT based preparations [3, 13, 14]. In contrary, a finding from south Africa concludes that, in population of HIV patients on treatment in resource-limited settings AZT-containing regimens appear to show a slightly protective than TDF-based regimens [15]. Despite, this evidence variation, its immunologic benefits unknown in an Ethiopian setting where patients generally present late, have high rates of TB and other infectious conditions [16]. Hence further investigation, particularly in health setups of the current setting‚ was warranted. Therefore, this study was aimed to provide additional information by making a head to head comparison of the two regimens in terms of immunologic benefits and associated risk factors in a teaching hospital of Ethiopia, one of the resource constrained settings.

## Methods

### Study area and period

The study was conducted at Jimma University specialized Hospital which is located in Jimma town; Jimma Zone, 346 km Southwest of Addis Ababa, Ethiopia. The hospital has separate ART clinic with about 7486 clients. The ART clinic provides HIV and TB treatment and care. Patient data from September 2012 to July 2014 was extracted from records from February 10, 2015 to March 10, 2015.

### Study design and population

A retrospective hospital based cohort study was conducted on adult patients who were on TDF and AZT based regimens and fulfilled inclusion criteria. The study was conducted by dividing the total sample into two groups: TDF and AZT groups.

### Inclusion and exclusion criteria

We included patients who were older than 14 years, have at least 6 months follow-up, whose records were legible and complete, and who have at least baseline and sixth month CD4 count. We excluded pregnant women from the study because there is significant pharmacokinetic change that is enough to impact the treatment outcome in this group of population. Those whose regimen was changed were also excluded from the study.

### Sample size and sampling techniques

Sample size determination was guided by the number of patients on TDF/3TC/NVP where only 70 patients fulfilled the inclusion criteria. Patients from other regimens were selected based on the size of this group to satisfy 1:1 ratio of AZT to TDF group. Therefore, frequency matching was used to select a total of 280 subjects (140 TDF group and 140 AZT group). Records were selected by simple random sampling technique using computer generated random numbers.

### Data collection

Data on demographic, clinical, laboratory, drug administered, comorbidities and adherence were collected by record review using English version checklist which was prepared after reviewing different relevant literatures. Baseline body mass-index of the subjects was calculated after collection of baseline height and weight of the patient from patients chart. Data from antiretroviral drugs sheet and patient information sheet were collected by pharmacists, while nurses collected data from ART intake and follow up forms.

### Data processing and analysis

Data were double entered into Epi-Data 3.1 and exported to STATA 13.1 for cleaning and analysis. Descriptive analyses were performed and results were presented by text, tables and charts. Bi-variate and multivariable mixed effect linear regression for repeated measurements were performed to assess adjusted effect of the ART regimen and identify additional predictors of CD4 recovery. Coefficient of mean CD4 count with 95% confidence intervals was used as measure of strength of association and P < 0.05 was considered to declare a statistical significance. Marginal analysis was also conducted to see the difference among specific regimen category.

## Results

**Fig. 1:**
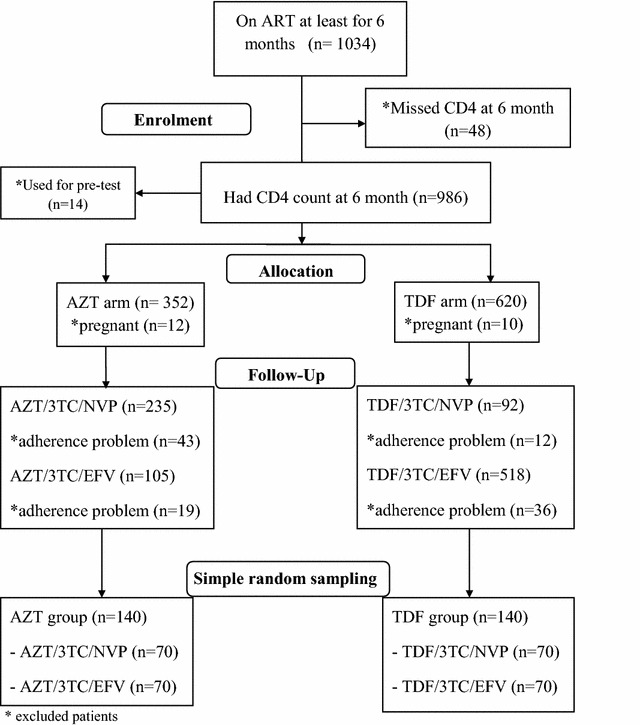
Sample recruitment diagram from cohort of patients on ART for at least 6 months at JUSH, September, 2012 to July, 2014

A total of 1034 patients were on antiretroviral therapy (ART) for at least 6 months during the study period. Nine hundred eighty six had complete CD4+ count at 6 month of treatment. Fourteen records were used for pre-test, 22 and 110 records were excluded because of pregnancy and regimen change respectively. Only 70 patients remained on TDF/3TC/NVP and this governed the sample selection (Fig. 1).

The cohort was followed for 2 years. The overall time the cohort was at risk was 539.39 years. The cohort contributed to a total of 2.74/100 and 2.72/100 person-years of follow-up for TDF and AZT groups respectively. The mean (SD) duration of follow up was 714.2 (69.6) and 708.8 (78.9) days (*P* = 0.753) for TDF and AZT groups respectively. The minimum follow up duration was 7.4 and 8.9 months for TDF and AZT groups respectively. Most of TDF (93.6%) and AZT (91.4%) groups completed their follow up, 5 (3.6%) TDF and 6 (4.3%) AZT groups died and 4 (2.9%) TDF and 6 (4.3%) AZT groups were lost for follow-up (*P* = 0.769).

### Baseline socio-demographic and behavioural characteristics

Majority of TDF (64.3%) and AZT (66.4%) groups were females. Majority of TDF (77.1%) and AZT (85.9%) groups were in the age group 25–45 years with mean (SD) of 32.3 (7.4) and 32.3 (9.2) years respectively. Majority of TDF (62.9%) and AZT (73.6%) groups had BMI ≥ 18 kg/m^2^ with mean (SD) of 19.7 (3.4) and 20.4 (3.0) respectively. Larger proportion of TDF (48.6%) and AZT (41.5%) groups were employed. Majority of TDF (69.3% and AZT (78.5%) groups were urban. Concerning alcohol use, most of TDF (80.7%) and AZT (72.9%) groups had history of alcohol use. Regarding between groups comparison of distribution of baseline socio-demographic and behavioural characteristics of patients, there was statistically significant difference between groups only in terms of religion (Table 1).

**Table 1:** Socio-demographic and behavioural characteristics for cohort of patients on ART for a least 6 months at JUSH, September 2012 to July 2014

Baseline socio-demographic and behavioural characteristics	Group		P value
	TDF group no (%)	AZT group no (%)	
Sex			
Male	50 (35.7)	47 (33.6)	0.706
Female	90 (64.3)	93 (66.4)	
Age			
<25	27 (19.3)	32 (25.9)	0.276
25–45	108 (77.1)	98 (85.9)	
>45	5 (3.6)	10 (7.2)	
Mean (SD)	32.3 (7.4)	32.3 (9.2)	0.977**
BMI			
<18.5	52 (37.1)	37 (26.4)	0.130
≥18.5	88 (62.9)	93 (73.6)	
Mean (SD)	19.7 (3.4)	20.4 (3.0)	0.062**
Educational level			
Illiterate	22 (15.8)	30 (21.4)	
Primary	48 (34.2)	58 (41.4)	0.089
Post-primary	70 (50)	52 (37.2)	
Residence			
Urban	97 (69.3)	110 (78.5)	0.077
Rural	43 (30.7)	30 (21.5)	
Occupation			
Employed	68 (48.6)	58 (41.5)	
Unemployed	46 (22.8)	55 (39.2)	0.446
Housewife	26 (18.6)	27 (19.3)	
Religion			
Orthodox	59 (42.1)	80 (57.1)	
Muslim	45 (32.1)	42 (30)	0.010*
Others	36 (25.8)	18 (12.9)	
Marital status			
Married	76 (54.3)	77 (55.0)	
Single	23 (16.5)	29 (20.7)	0.207
Divorced	33 (23.5)	21 (15.1)	
Widowed	8 (5.7)	13 (9.2)	
Alcohol use			
Yes	113 (80.7)	102 (72.9)	
No	27 (19.3)	38 (27.1)	0.120

* Statistically significant at P value 0.05 cut off point

** T test

### Baseline clinical characteristics

Majority of TDF (65.7%) and AZT (53.9%) groups had baseline CD4+ count <200 cells/mm^3^ with mean (SD) of 164.64 (83.36) and 175.21 (89.14) respectively. There was statistically significant difference between groups in terms of baseline CD4 count. Leading percentage (33.6%) both TDF and AZT groups were in WHO clinical stage III and II respectively. Most of TDF (78.6%) and majority of AZT (56.4%) groups had working functional status and there was statistically significant difference between groups. Twenty (15.3%) and 17 (12.1%) of patients were treated for TB from TDF and AZT groups respectively. CPT was prescribed for most of (87.9%) and nearly all of (92.14%) patients in TDF and AZT groups respectively, whereas INH was prescribed for only 26.4 and 21.5% of patients in TDF and AZT groups respectively (Table 2).

**Table 2:** Baseline and on follow up clinical characteristics for cohort of patients on ART for a least 6 months at JUSH, September 2012 to July 2014

Clinical characteristics	TDF group no (%)	AZT group no (%)	P value
Baseline CD4+ count			
<200	92 (65.7)	74 (53.9)	0.029*
≥200	48 (34.3)	66 (47.1)	
Mean (SD)	164.64 (83.36)	175.21 (89.14)	0.307**
Baseline WHO clinical stage			
I	32 (22.9)	36 (25.7)	
II	46 (32.9)	47 (33.6)	0.928
III	47 (33.6)	43 (30.7)	
IV	15 (10.6)	14 (10)	
Baseline functional status			
W	110 (78.6)	79 (56.4)	
A	24 (17.1)	56 (40.0)	0.000*
B	6 (4.3)	5 (3.6)	
TB Treatment			
No	120 (84.7)	113 (87.9)	0.773
Yes	20 (15.3)	17 (12.1)	
Prophylaxis			
CPT+ INH	37 (26.4)	30 (21.5)	
CPT alone	86 (61.4)	99 (70.7)	0.231
Neither	17 (12.2)	11 (7.8)	

* Statistically significant at P value 0.05 cut off point

** T test

### Immunologic outcome (CD4+ change)

**Fig. 2:**
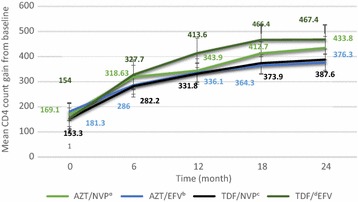
Average gain in CD4+ count taken every 6 month for a cohort of patients on ART for at least 6 months at JUSH, September, 2012 to July, 2014. Missing values per each regimen at 12, 18 and 24 months respectively: ^ a^(2, 7, 11); ^b^(11, 3, 2); ^c^(7, 1, 9); ^d^(5, 12, 4)

The mean change in CD4+ over study period is shown by Fig. 2. The overall mean (SD) CD4+ has shown greater improvement among TDF [321.7 (164.8)] than AZT group [299.4 (126.1)]. If each regimen is considered separately, the maximum and minimum mean CD4+ count gain at any given time was attained by TDF/3TC/EFV and AZT/3TC/EFV groups respectively (Fig. 2).

At 6 month, the overall proportion of sub-optimal CD4+ response (net CD4+ gain <50 cells/µl) was 23.95% (30% TDF/EFV, 28.3% AZT/EFV, and 23.8% TDF/NVP). Earlier at the initiation of ART, the CD4+ count showed a linear trend but it becomes more flat after 18th month with a very minimal gain irrespective of the regimen.

### Predictors of CD4+ change

Considering consecutive CD4+ count as an outcome variable, multilevel mixed effect linear regression was performed (Table 3). The slope of random-effect multiple linear regression was used to interpret the overtime change in CD4+ count attributed to the predictor variables.

**Table 3:** Random-effect linear regression analysis of trend of CD4+ count (slope, cells/mm^3^/6 month) at JUSH, September 2012 to July 2014

Variable	Unadjusted		Adjusted	
	B [95% CI]	P value	B [95% CI]	P value
Sex				
Male	0		0	
Female	52.373 [21.22, 84.24]	0.001	33.86 [33.11, 43.24]	0.013
Age				
<25	0		0	
25–45	−53.15 [−90.03, −16.27]	0.005	−27.32 [−59.3, 4.66]	0.094
>45	−120.87 [−191.24, −50.49]	0.001	−66.19 [−126.68, −5.70]	0.032
BMI				
**<**18.5	−48.08 [−80.42, −15.74]	0.004	−32.77 [−60.16, −4.27]	0.011
≥18.5	0		0	
Educ. level				
Illiterate	−0.5 [−42.86, 41.86]	0.982		
Primary	14.42 [−19.59, 48.44]	0.406		
Post-primary	0			
Residence				
Urban	0			
Rural	−12.35 [−47.18, 22.54]	0.488		
Occupation				
Employed	0	0.936	0	0.796
Unemployed	−1.40 [−32.23, 32.44]	0.015	3.96 [−26.04, 33.95]	0.293
House wife	51.50 [10.2, 92.80]		21.35 [−18.42, 61.12]	
Marital status				
Single	30.25 [−11.18, 71.68]	0.152	40.45 [0.52, 80.37]	0.069
Married	0	0.525	0	
Divorced	13.06 [−27.17, 53.29]	0.513	6.42 [−32.27, 45.11]	0.745
Widowed	−19.89 [−79.56, 39.77]		−2.33 [−55.27, 50.6]	0.931
Religion				
Orthodox	0		0	
Muslim	22.91 [*−*12.00, 57.83]	0.198	20.83 [−8.86, 50.53]	0.166
Other	0.21 [*−*40.70, 41.13]	0.992	−12.09 [−46.89, 22.71]	0.496
Alcohol				
Yes	*−*26.1 [*−*62.15, 9.94]	0.156	8.48 [−22.88, 39.83]	0.596
No	0		0	
ART regimen				
TDF	11.58 [−18.98, 42.15]	0.458	34.08 [7.80, 60.35]	0.027
AZT	0		0	
Baseline CD4+ count	0.97 [0.80, 1.15]	0.000	0.879 [0.70, 1.06]	0.000
WHO stage				
I	0			
II	−16.76 [−57.59, 24.06]	0.421		
III	−23.17 [−64.32, 17.97]	0.270		
IV	−29.27 [−86.35, 27.81]	0.315		
TB (treatment)				
No	0		0	
Yes	−48.18 [−85.37, 5.00]	0.081	−20.74 [−59.05, 17.57]	0.289
Prophylaxis				
CPT+ INH	0		0	
CPT alone	−29.40 [−65.65, 6.85]	0.112	−10.34 [−40.57, 19.88]	0.502
Neither	−40.89 [−97.93, 16.16]	0.160	−22.3 [−70.33, 25.73]	0.363

Accordingly, the average gain in CD4+ count achieved at every visit among the cohort was 38 cells/mm^3^ (B = 38.18, 95% CI [33.11,43.24], P < 0.001) with the conditional correlation coefficient of 64.9%, i.e. 64.9% of the variability in CD4+ count between two visits was explained by unobserved patient specific factors. Among these, 38.99% of the variation in CD4+ change was explained by differences in the regimens where as 25.34% of the variation is attributed to other regression variables (P < 0.001), showing an important heterogeneity between patient groups.

In the overall analysis, keeping other factors constant, older age was found to be one of the negative predictors of CD4+ count change. So younger patients (age <25 years) were found to have +66 gain in CD4+ count every 6 months as compared to those aged ≥45 years (B = −66.19 95% CI **[**−126.68, −5.70], P = 0.032).

Among the predictor variables, female sex was strongly associated with progressive CD4+ count gain at each visit. So, females had +39 CD4+ cells advantage over time (B = 33.86, 95% CI [33.11, 43.24], P = 0.013) as compared to males. Patients with BMI ≥ 18.5 kg/m^2^ had a significantly higher CD4+ change (+32 cell/mm^3^) over time than those who had BMI < 18.5 kg/m^2^ (B = −32, 95% CI [−60.16, −4.27], P = 0.011).

Baseline CD4+ count was also another independent predictor for CD4+ advantage over time (β = 0.879, 95% CI [0.70, 1.06], P < 0.001). More importantly, patients in TDF group had a significant CD4+ count gain per visit compared with their AZT counterparts (B = 34.08, 95% CI [7.80, 60.35], P = 0.027).

In this study, other baseline patient factors such as WHO clinical stage, TB treatment, educational status, place of residence, religion, occupational status, marital status, alcoholic use and prophylaxis had no significant association with CD4+ change.

### Marginal analysis

To predict the expected change in CD4+ count in both groups at the end of treatment period, post-estimation marginal analysis was conducted.

In TDF group, the predicted mean CD4+ count changes were 347.65 and 295.73 cells/mm^3^ for patients treated with TDF/3TC/EFV and TDF/3TC/NVP respectively. In AZT group, the predicted mean CD4+ count changes were 319.11 and 281.54 cells/mm^3^ for patients treated with AZT/3TC/NVP and AZT/3TC/EFV respectively. These figures were exactly the expected increase in CD4+ count associated with each regimen and has a crucial clinical implication in guiding clinicians to choose which regimen to initiate on as the role of good immunologic recovery in treatment of HIV infection is multidimensional. So, this section of analysis (Table 4) clearly showed that the overall outcome was better in TDF than AZT group.

**Table 4:** The predicted mean CD4+ change of patients treated with AZT and TDF based regimens at JUSH, September 2012 to July 2014

Status	Status	Delta-method		t	[95% CI]	P value
		Margins	Standard error			
Unexposed	AZT3TC/NVP	319.11	19.34	16.50	281.02,357.20	P < 0.001
	AZT/3TC/EFV	281.54	18.39	15.31	245.31,317.77	P < 0.001
Exposed	TDF/3TC/NVP	295.73	18.39	16.08	259.50,331.96	P < 0.001
	TDF/3TC/EFV	347.65	18.39	18.90	311.42,383.89	P < *0.001*

## Discussion

Analysis of every 6 month mean CD4+ gain showed that the maximum gain in mean CD4+ count was attained by TDF/3TC/EFV followed by AZT/3TC/NVP and TDF/3TC/EFV at any given time in the course of therapy. AZT/3TC/EFV had the least immunologic recovery over the entire treatment course. The CD4+ count showed a linear trend but became more flat after 18th month with a very minimal gain irrespective of the regimen. The overall prevalence of sub-optimal CD4+ response was found to be 67 (23.95%). At first 6 months, majority of sub-optimal immunologic responders belonged to TDF/3TC/EFV (30%) and lowest proportion of sub-optimal immunologic responder was observed among AZT/3TC/EFV (17.9%).

Our finding of mean CD4+ recovery was in agreement with a randomized multi-centre open-label study by Gallant et al. [9], where a maximum immunologic response was achieved by TDF/3TC/EFV (270 cells/mm^3^) followed by AZT/3TC/EFV (237 cells/mm^3^) at 96 weeks. Another finding from Nigeria indicated that TDF/3TC/NVP is much more inferior (208 cells/mm^3^) to AZT/3TC/NVP (221.1 cells/mm^3^) at 12 months of therapy [17]. The difference in the outcome when TDF/3TC is combined with EFV and NVP may be due to negative pharmacokinetic or pharmacodynamics interaction between this NRTI backbone and NVP. The finding from the marginal analysis also indicated that change in mean CD4+ count is significantly higher in TDF based EFV regimen.

The bi-phasic CD4+ count response shown in our finding was also reported by other findings [18, 19] as a rapid increase of memory CD4+ cells (a high CD4+ count slope) in the first several months after treatment initiation succeeded by a slow increase in naive CD4+ cells (smaller slope compared to the initial several months). The linear trend in CD4+ increment at early phase of of therapy became flat with minimal CD4+ gain latter after 18th month of treatment irrespective of the regimen used. So “when will target CD4+ count (500–800 cells/mm^3^) be attained after initiation of ART?” is the question to be addressed by further study.

Immunologic response after 6 months of ART indicates a favourable clinical outcome in HIV-1 infected patients regardless of virologic response [20]. Several studies has reported that as many as 8–40% of patients on ART do not show a significant increase in CD4+ cell count despite viral suppression [18, 21, 22]. Our finding, in general, is almost consistent with these studies.

To these days, studies are unable to justify the impact of ART regimen on sub-optimal immunologic recovery. So, it is not surprising that most of these patients in this study were from TDF based regimens. As the recovery of the CD4+ T-cell count is hindered by several patient and viral factors, including: residual viral replication, impaired thymic function, advanced age, enhanced T-cell activation and apoptosis, genetic variations, baseline anaemia and poor adherence [18, 21, 23, 24], our finding might not quest the efficacy of TDF based regimens in resource limited settings, even though it needs a further workup with adequately powered and methodological high quality study.

Study by Mauro et al. [25] has confirmed older age as a key independent predictor for sub-optimal immunologic response as thymus activity decreases with age [26]. This finding agreed with our result where older age was statistically significant negative predictor of CD4+ gain on multiple regression.

The pre-treatment CD4+ count in relation to sub-optimal immunologic response, however, is controversial as some literatures favour higher baseline CD4+ (>200 cells/mm^3^) [26] and explained it as “starting treatment at higher CD4+ cell counts limits the scope for further increases”. Other literatures favoured lower baseline CD4+ count (<200 cells/mm^3^) and this is biologically plausible given that a low nadir pre-treatment CD4+ cell count is thought to be suggestive of more extensive depletion of CD4+ cells in the gut-associated lymphoid tissue during acute HIV infection, and may be delayed or refractory to reconstitution with ART [27].

As this study has short comings including absence of viral marker measurement to conclude the rate of sub-optimal immunologic recovery among the patients, further study with quality design is needed as lack of knowledge about this subgroup may contribute to inadequate clinical management and current HIV treatment guidelines do not provide specific applicable guidance [18].

The overall random effect linear regression analysis had pointed out that, baseline BMI, sex, age, baseline CD4+ count, and exposure to TDF based regimen were independent predictors for CD4+ change over time. The marginal effects of each regimens confirmed that the immunologic outcome associated with TDF based EFV preparations was more convincing and made it the golden regimen to be utilized in this setup (margins = 347.65 cells/mm^3^/, *P* < *0.001*) followed by AZT/3TC/NVP (m = 319.11 cells/mm^3^, *P* < *0.001)*and TDF/3TC/NVP (m = 295.73, P < 0.001). However, AZT/3TC/EFV had lowest predicted change in CD4+ count (m = 281.54, P < 0.001). This implies that this regimen has minimal immunologic response and its clinical utilization need to be reconsidered.

Females had greater CD4+ improvement over time. Accordingly, every visit of female patients was associated with the average CD4+ count of 39 cells/mm^3^ (*β* = 33.86 [33.11, 43.24], P = 0.013). Similar study from Lao Democratic Republic strengthen this finding [28]. But it was inconsistent with study from Asia [19] probably due to differences in study setup and sample size (1676 versus 280), where females contribute only 26% of the sample analysed.

The impact of age on immunologic recovery was well described [26] and this study had found concordance with previous findings. Accordingly, the CD4+ gain attained by younger (<25 years) patients was +66 cells/mm^3^ as compared to those older than 45 years of age (β = −66.19, 95% CI [−126.68,−5.70], P = 0.032). The finding is consistent with the studies from Asia [19] and Ghana [29] and which reported the inverse relationship between age and CD4+ gain. This is mainly due to failure of cellular expansion or non-sustained cell survival in the periphery or age related central degeneration of thymus function as patients become older [18].

In the cohort, those with BMI ≥ 18.5 kg/m^2^ had a better immunologic outcome and each visit was associated with 32 cells/mm^3^ of CD4+ count advantage as compared to patients with BMI < 18.5 kg/m^2^ (β = −32.22, 95% CI [17.23,51.85], P < 0.001). This is concurrent with the study by Bastard et al. [ [28].] in which BMI > 18 kg/m^2^ was reported to have a protective effect for CD4 count increment at 9 months of therapy (HR = 0.39, 95% CI 0.25–0.60). This implies that higher BMI is a sign of good nutritional status and it is fertile ground for better immunologic revival.

The baseline CD4+ count was another positive predictor for successful immunologic revival. So, a patient has a 0.879 CD4+ cell count advantage over time, for every count higher in initial CD4+ count, when compared to his/her counterpart (*B* = 0.879 95% CI [0.70, 1.06]*, P* < 0.001). This finding is consistent form study by Lawrence et al. [27] and Mustapha et al. [29] in which higher baseline CD4+ count was associated with good immunologic outcome. This might be due to less extensively depleted immune system will be boosted easily after initiation of ART. The overall clinical and immunological findings were suggestive of better outcome of ART if initiated at higher CD4+ count. This finding also agrees with the recent WHO ART guidelines [30] which described the initiation of ART irrespective of WHO stage and CD4+ count in adolescents and adults.

In contrary, the finding disagrees with some studies showing poor immunologic recovery including discordant responders when ART was commenced at higher CD4+ count [31]. This may be due to the goal CD4+ in HIV patients (500 cells/mm^3^) [32] can be attained immediately in those with higher baseline CD4+ and further increment could be impossible. The reason for the deviation might be due to differences in sample size and set-up. The multicenterity of the previous study might also contribute for the difference.

From this study, patients randomized to TDF group had a significant CD4+ count advantage per visit relative to patients randomized to AZT group (*B* =+34.08, 95% CI [7.80, 60.35], P = 0.001). This study is consistent with most of the previous findings that described TDF based regimens with better immunologic outcome [9, 14, 31, 32].

## Conclusions

The results of this study suggest that TDF based regimens especially, TDF/3TC/EFV had excellent immunologic recovery followed by AZT based NVP. Since aged patients, those with baseline CD4+ count <200 cells/mm^3^ and patients with pre-treatment BMI < 18.5 were poor immunologic responders, they need special attention while delivering care and treatment. However, the prevalence of sub-immunologic recovery among the TDF users in the resource constrained settings needs to be assessed further.
